# Outcome of living kidney donors according to national data of turkish organ and tissue information system

**DOI:** 10.1186/2197-425X-3-S1-A901

**Published:** 2015-10-01

**Authors:** A Kapuağası, A Özcan, İ Şencan, MA Aydın, M Öztürk, Z Uzundurukan, AH Elhan, H Başar, Ç Kaymak

**Affiliations:** General Directorate of Health Services, Ministry of Health, Organ, Tissue and Transplantation Department, Ankara, Turkey; Ankara Training and Research Hospital, Ministry of Health, Department of Anesthesiology and Reanimation, Ankara, Turkey; General Directorate of Health Services, Ministry of Health, Ankara, Turkey; Faculty of Medicine, Ankara University, Department of Biostatistics, Ankara, Turkey

## Introduction

Live kidney transplantation has several advantages than cadaveric-kidney transplantation and is still frequently performed. When assessed in terms of donor consent and religious concepts, there is a negative relationship between these terms and live kidney transplantation. However, there is little information about renal disease, social and psychological problems of donors after donation.

## Objectives

We assessed the outcome of living kidney donors after donation in National Data of Turkish Organ and Tissue Information System.

## Methods

Outcome of living kidney donors between years 2011-2014 was reviewed. Age and gender of the patients were recorded. Mean follow-up time and 6 months, 1, 2 and 3 years survival of the patients were identified. Kaplan-Meier method was used to calculate the survival rates.

## Results

The number of live kidney transplantation is 9473 between years 2011-2014 in Turkey. The mean age of the donors was 49.04 ± 12.80 (mean ± SD), and 43 % (4077) of the donors were male and 57 % (5396) were female. The mean follow-up time of the living kidney donors was 27.28 ± 13.83 (mean ± SD) and 6 months, 1, 2 and 3 years survival were 99.9 %; 99.9 %; 99.9 % and 99.9 %, respectively.

## Conclusions

Live kidney donation is a frequent practice in our country and organized by scientific committees. Survival times of living kidney donors in our country are higher compared to results in the literature. In respect to high rates of live kidney transplantation in our country, potential kidney donors should be evaluated in detail before donation and there is a need for long term follow-up and consulting service after donation.
